# A case report: Clozapine‐induced leukopenia and neutropenia after mRNA COVID‐19 vaccination

**DOI:** 10.1002/npr2.12238

**Published:** 2022-02-15

**Authors:** Tomoyuki Imai, Sho Ochiai, Takehiro Ishimaru, Hayato Daitoku, Yusuke Miyagawa, Ryuji Fukuhara, Shuken Boku, Minoru Takebayashi

**Affiliations:** ^1^ 157728 Department of Neuropsychiatry Kumamoto University Hospital Kumamoto Japan; ^2^ 157728 Comprehensive Clinical Education, Training and Development Center Kumamoto University Kumamoto Japan; ^3^ 36921 Minamata City General Hospital and Medical Center Minamata Japan; ^4^ Department of Neuropsychiatry Faculty of Life Science Kumamoto University Kumamoto Japan

**Keywords:** clozapine, COVID‐19 vaccine, leukopenia, neutropenia, schizophrenia

## Abstract

Clozapine is an atypical antipsychotic used for treatment‐resistant schizophrenia and is known to cause serious side effects, such as leukopenia and neutropenia. We encountered the case of a 44‐year‐old female patient with a good response to clozapine, who experienced inflammatory reaction and cytopenia after coronavirus disease 2019 (COVID‐19) vaccination. Soon after clozapine discontinuation, the inflammatory reaction resolved, and cell counts recovered. There are only a few reports on the interaction between clozapine and COVID‐19 vaccine. Our findings suggest that caution is required when a patient who is receiving clozapine scheduled for COVID‐19 vaccination, owing to the possibility of cytopenia. Moreover, blood tests and the measurement of clozapine concentration should be performed before and after the inoculation to ensure patient safety.

## Abstract 2

We encountered the case of patient with treatment‐resistant schizophrenia and a good response to clozapine, who experienced inflammatory reaction and cytopenia after COVID‐19 vaccination. Soon after the drug discontinuation, the inflammatory reaction resolved, and cell counts recovered.

## Graphical abstract

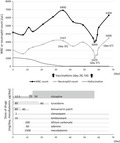

## 1 CASE

The patient was a 44‐year‐old Japanese woman with symptoms of schizophrenia, particularly hallucination and avolition. Almost all atypical antipsychotics, such as olanzapine (15 mg), brexpiprazole (2 mg), and lurasidone (80 mg), were ineffective in combating the symptoms or were poorly tolerated; therefore, the dosages of these drugs were reduced, and they were discontinued before starting clozapine.

**FIGURE 1:**
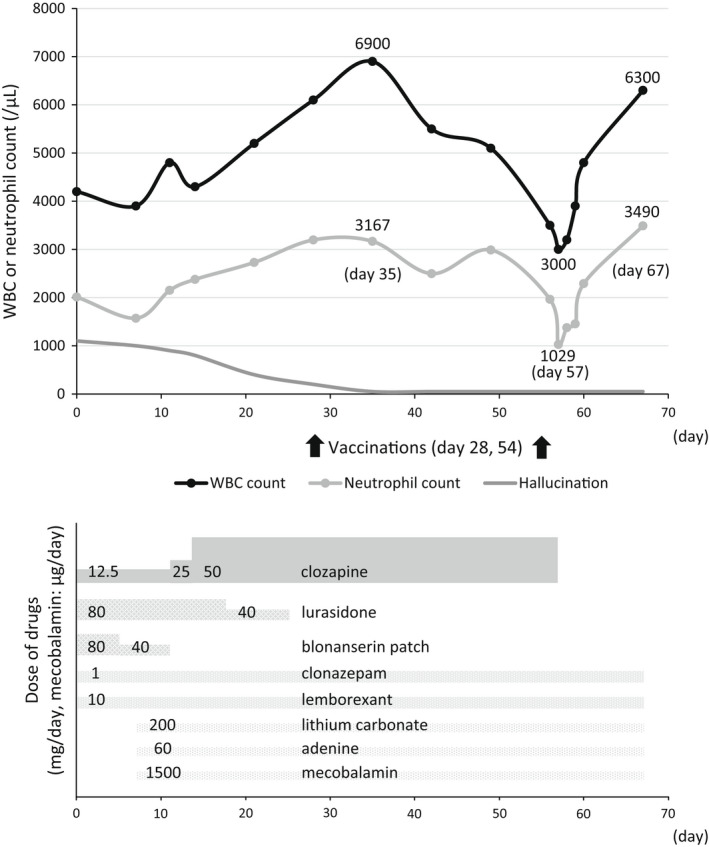
Clinical course of the patient

After blood testing (day 0), treatment with 12.5 mg clozapine tablets, which were taken orally after every evening meal, was started (day 1). A week later (day 7), the patient's white blood cell (WBC) count had decreased from 4790 to 4030/μL, and the neutrophil count had decreased from 2012 to 1572/μL. On the same day, lithium carbonate (200 mg), adenine (60 mg), and mecobalamin (1500 μg) were added to her drug regimen to increase her WBC count. As expected, her WBC count normalized a few days later. After the clozapine dose was increased to 50 mg (Figure 1), some of the patient's previous medications were tapered (80 mg lurasidone in approximately one month and 80 mg blonanserin patch in 10 days). In addition, 1 mg clonazepam and 10 mg lemborexant were continued. The WBC count increased to 6900/μL and the hallucination reduced.

On day 28, the BNT162b2 mRNA COVID‐19 vaccine (tozinameran, Pfizer‐BioNTech) was injected into the left deltoid muscle. After the injection, her WBC count gradually decreased. On day 54, she received the second shot of the vaccine. On the next day, the patient experienced fatigue and pain in the left parotid gland and had a temperature of 37.5°C, and her C‐reactive protein (CRP) level was slightly elevated to 10.1 mg/L. On day 56, her WBC and neutrophil counts further decreased. On the following day, her WBC and neutrophil counts were 3000 and 1029/μL, respectively. A decision was made to stop the clozapine. Soon after the discontinuation of clozapine, her WBC count and the ratio of neutrophils to other leukocytes normalized.

The other side effects of clozapine, such as slight drowsiness and hypersalivation, also subsided. The patient did not have severe acute respiratory syndrome coronavirus 2 (SARS‐CoV‐2) infection, and her glucose tolerance and blood glucose level were unaffected throughout the process. We were unable to measure her blood concentration of clozapine in our facility.

## 2 DISCUSSION

In this case, there are three possibilities for the cytopenia. First, the inflammatory reaction caused by COVID‐19 vaccination probably inhibited cytochrome P450 1A2 activity, suppressing clozapine metabolism and elevating the blood level of the medication.^1^ A previous research indicated that clozapine level is influenced by inflammatory inhibition of cytochrome P450 1A2 activity.^2^ The patient would have felt better and had the hallucination resolved by a very low dose (50 mg) of clozapine; the results suggest that she had a poor metabolism of the drug, and that the concentration of the medication might have increased after the vaccination. However, this is only a supposition because, as mentioned above, clozapine concentration was not measured.

Second, an indeterminate immune response to the vaccination might have occurred, and the response may have induced cytopenia in the presence of clozapine. A report about severe agranulocytosis as a side effect of clozapine use after influenza vaccination^3^ suggested the existence of immunological mechanisms of clozapine‐related leukopenia.

Lastly, the cytopenia could have been caused by a gradual increase in clozapine concentration. From the time of initiation of clozapine therapy, there was a tendency for abnormal blood cell counts. The secondary decrease in WBCs and neutrophils possibly coincided with the time of mRNA vaccination. A limitation of this case report is that clozapine was started just before the vaccination, and the duration of the administration was short, including the period that is most associated with agranulocytosis (18 weeks).

The relationship between COVID‐19 vaccine and granulocytopenia in patients receiving treatment with clozapine is uncertain; further case studies are needed to elucidate this relationship. Regarding the clinical use of clozapine, it is difficult to replace clozapine with other antipsychotics. In Japan, the guidelines since June 2021 recommend that clozapine may be carefully restarted if hematological findings improve.^4^

In conclusion, psychiatrists should be aware of the risk of side effects, including cytopenia, when patients who are receiving treatment with clozapine are to be vaccinated. Therefore, blood cell count, and if possible, plasma concentration of clozapine should be measured before and after the inoculation for close monitoring.

## CONFLICT OF INTEREST

The authors have no conflicts of interest to declare.

## AUTHOR CONTRIBUTIONS

All authors contributed to the writing of the paper and approved the final manuscript.

## INFORMED CONSENT

Informed consent from the patient was acquired at the time of the publication of this report.

## Data Availability

Data sharing is not applicable for this article as no datasets were generated or analyzed during the current study.
